# Surgical Approaches to Parapharyngeal Space Tumors: An Example and Review of the Literature

**DOI:** 10.1155/2021/3536145

**Published:** 2021-08-30

**Authors:** Souheil Jbali, Amira Khaldi, Slim Touati, Said Gritli

**Affiliations:** ENT Department, Salah Azaiez Institute of Cancer, Medicine University, Tunis, Tunisia

## Abstract

Parapharyngeal space tumors (PPS) are rare. They represent less than 1% of all head and neck tumors. They are of various histologies. Pleomorphic adenomas originating from the parotid (prestylian parapharyngeal space) are the most frequent. We report the case of a 50-year-old patient treated for a pleomorphic adenoma (PA) of PPS. His initial complaints were apnea and dysphagia. The correct diagnosis was preoperatively suspected by magnetic resonance imaging (MRI). The surgery was carried out using two approaches: transoral and cervical transparotid approaches. Definitive histology was consistent with encapsulated pleomorphic adenoma. In the present work, we reviewed clinical, radiological, and histological features of parapharyngeal space tumors. We tried to summarize the common surgical approaches. The chosen approach is, in fact, scheduled taking into account several parameters including tumor volume and purpose of the surgery.

## 1. Introduction

The parapharyngeal spaces are located below the skull base, laterally to the upper part of the oropharynx and separated from the cervical region by the hyoid bone plane. The fascia running posteriorly from the styloid process to the tensor veli palatini muscle divides the parapharyngeal space into prestyloid (anterior) and poststyloid (posterior) compartments. The poststyloid compartment contains the internal carotid artery, the internal jugular vein, cranial nerves IX, X, XI, and XII, the cervical sympathetic chain, and lymph nodes. Poststyloid tumors can arise from each of these structures, and equally these structures are at risk during surgery of the parapharyngeal space. The prestyloid compartment is composed largely of fat, the retromandibular portion of the parotid gland deep lobe, and lymph nodes.

Tumors of the PPS account for 0.5% to 1% of all head and neck masses [1, 2]. Due to this low prevalence, the literature on this topic is often limited to single-center case reports or case series. A systematic review of 1293 cases reported over 25 years was recently published [3]. Malignancies represent less than 25% of all tumors located in these parapharyngeal spaces and are mainly of salivary origin [4]. Salivary tumors account, indeed, for about 42% of PPS tumors. Neurologic and vascular tumors that are commonly located in the retrostylian (poststyloid) parapharyngeal space are the second most frequent histologic types [2].

## 2. Case Report

A 50-year-old male patient was admitted to our clinic. He complained of a recent increase in snoring, apnea attacks, and swallowing difficulties. There was no mass on the neck palpation. Physical examination revealed a bulging of the anterior pillar of the palatal tonsil resulting in slight obstruction of the upper airway.

**Figure 1:**
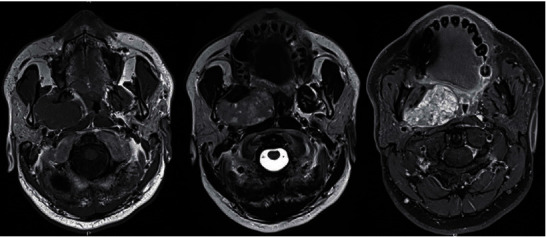
Facial MRI in axial sections weighted T1, T2, and T1 injected (from left to right): prestyloid parapharyngeal tumor independent of the parotid deep lobe with heterogeneous T2 hypersignal and enhancement after gadolinium injection.

The patient underwent neck MRI with gadolinium injection (Figure 1). It revealed a well-defined 52 × 38 mm tumor of the right PPS, which was hypointense on T1-weighted sequences and hyperintense on T2. There was a displacement of the right oropharyngeal wall medially. It was independent of the deep lobe of the parotid. These MRI features were consistent with PA with possible focal areas of malignant degeneration in diffusion-weighted imaging (DWI) sequences.

The tumor was not extirpable through the transoral approach alone. A combined approach associating cervico-parotid and transoral incisions was therefore necessary. This approach allowed the exposure of the facial nerve avoiding its damage.

The postoperative course was uneventful apart from House's grade III facial palsy which regressed using corticosteroids.

Definitive histology was consistent with encapsulated pleomorphic adenoma.

NB: the patient has given his consent for the MRI images to be used for research and the case to be published.

## 3. Discussion

The parapharyngeal space is a complex region of the face. Tumors that develop in this region include nearly seventy different histologic subtypes [2]. More than eighty percent of these lesions are benign [3].

Salivary gland tumors are the most common primary lesions followed by neurogenic lesions. They represent 45% and 40% of all PPS tumors, respectively [1–5].

As most prestyloid tumors arise from salivary glands (parotid and accessory salivary glands), pleomorphic adenoma is the most common histologic type accounting for 34% of all primary parapharyngeal lesions and 65% of all salivary gland lesions [1–5].

Malignant salivary gland lesions account for 23% of all salivary gland lesions (adenoid cystic carcinomas and muco-epidermoid carcinomas are the most common) [2].

Parapharyngeal space lesions extend in both medial and inferior directions. The consequence is an asymmetric intraoral swelling, which is typically smooth and not ulcerated. Maran et al. [6] stated that parotid lesions distort typically the tonsil, while neurogenic lesions distort the posterior tonsil pillar. Downward growth can manifest as a submandibular tumefaction.

Extensive tumors to the poststyloid compartment can encroach upon the cranial nerves IX, X, XI, and XII and cause partial or full paralysis, which was seen in 17% of all cumulative cases reported by Kuet et al. [3].

Other symptoms have been described: they include dysphagia and dysphonia reported in 11% and 9%, respectively [2]. Superior extension can compress the Eustachian tube leading to middle ear effusions and consequent hearing loss (5%) [6].

Some symptoms should alert the clinician to the possibility of malignancy. They include referred otalgia, facial pain, trismus, and cranial nerve deficiency [3].

MRI is, actually, the imaging technique of choice for the PPS tumors. Accurate in 95% of cases [7], it allows delineation of all PPS masses and precise its relationship to the surrounding tissues. Normal anatomy and tumor-fat interface are best studied on T1-weighted sequences, whereas tumor margins and tumor-muscle interface on T2-weighted sequences [7].

Accurate PPS tumor locations (pre- or poststyloid) may be guessed by studying PPS fat or internal carotid artery displacements. Fat is pushed anteriorly and medially in prestyloid tumors, forward and laterally in those developing in the poststyloid space [7].

Some tumors have particular descriptions. Then, paragangliomas have a classic salt and pepper appearance, due to vascular flow voids on both T1- and T2-weighted sequences. Schwannomas, which arise from the poststyloid compartment, tend to be homogeneous, with moderate to high signal on T2-weighted acquisitions. Prestyloid neoplasms, which originate predominantly from the parotid deep lobe and sometimes from ectopic salivary glands, have similar signals to schwannomas. They are distinguishable according to the internal carotid artery displacement, which is postero-medial in prestyloid lesions and antero-medial in poststyloid ones [8].

Otherwise, deep lobe parotid tumors may be differentiated from accessory salivary gland tumors by the preservation of the fat interface between the tumor and the parotid on T1-weighted sequences in accessory salivary gland tumors [7, 8].

In addition to clinical features, malignancies of the PPS may be suspected on MRI on the following radiological characteristics: invasion of surrounding tissues, bone destruction or hyposignal of the bone marrow on T1-weighted sequences, irregular tumor margins, and presence of lymphadenopathies [7, 8].

Regarding vascular tumors (vagal paragangliomas, carotid body tumors, etc.), preoperative angiography is usually recommended [7, 8].

Surgery remains the first and the main therapeutic option for PPS tumors. This surgery is aimed at complete tumor excision with minimal morbidity. The choice of approach is guided by tumor size, suspicion of malignancy, tumor's superior extension near the skull base, and relationship to major neurovascular structures.

As for facial bone tumors, three-dimensional (3D) reconstruction can be suggested as a useful and accurate method for volumetric evaluation of PPS tumor extensions [9]. This reconstruction requires a software supplement to the cross-sectional imaging devices (CT scan and MRI), and we believe that its contribution remains limited.

Three external surgical approaches are suitable for the majority of PPS tumors [1–3, 5].

The cervical approach is the most commonly used. It is very useful for direct access to the inferior portion of the PPS, good visualization of cranial nerves, and control of vessels. This cervical approach is often associated with parotid surgery and facial nerve dissection particularly in tumors including the parotid deep lobe or minor salivary glands.

A mandibulotomy associated with cervical approaches is indicated for large, recurrent, or malignant tumors requiring maximum exposure and control of large vessels (external carotid artery in particular). It is also indicated for tumors highly located in the PPS as they necessitate distal control of the internal carotid artery at the skull base.

The transoral approach is a less invasive procedure. It is indicated for accessible small and benign tumors of the prestyloid region or for biopsy. It may require an associated tonsillectomy. The disadvantages of this approach are absence of direct visualization of great vessels and the higher risk of tumor rupture. When used alone, it may result in incomplete tumor removal, uncontrollable hemorrhage, and possible poststyloid nerve injuries.

Recently, endoscopic and robotic approaches have been widely applied in head and neck surgery. Even for PPS tumors, some authors proposed a combined transcervical and video-assisted minimally invasive approach, using a kind of optics called Hopkins telescopes [10]. This video assistance seems interesting. It allows a larger vision and better control of the vasculo-nervous structures. It also helps to control hidden regions to visual inspection in open surgery like in otoendoscopy.

**Figure 2:**
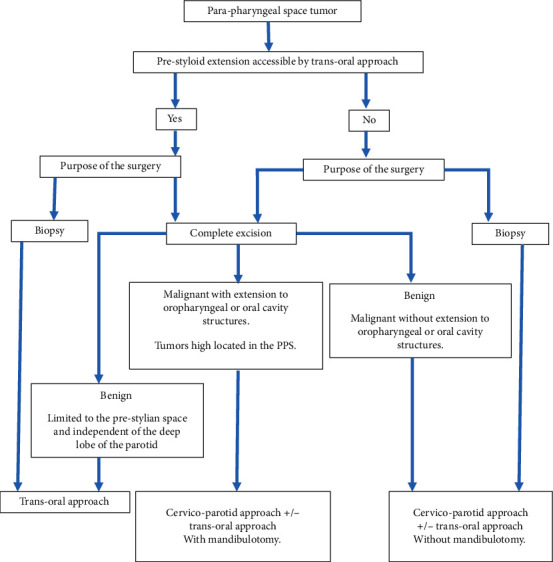
Strategy of choice of the surgical approach in parapharyngeal space tumors.

Finally, we tried to propose an algorithm that can help choose the appropriate surgical approach of the parapharyngeal space tumors (Figure 2).

## 4. Conclusion

Parapharyngeal space neoplasms are rare and mostly benign. Their location and extensions must be clearly specified before any treatment decision. Surgery is aimed at removing the entire tumor with minimal morbidity. For the majority of extirpable tumors, a cervical or cervico-parotid surgical approach is sufficient. An associated mandibulotomy is indicated for infiltrating malignant lesions requiring improved access. Care must be taken, however, to watch out for postoperative complications, in particular lesions of the postcricoid region nerves.
